# Evaluating ChatGPT as a Standalone Tool for Patient Education: A Review of Frequently Asked Questions by Patients With Chronic Obstructive Pulmonary Disease

**DOI:** 10.7759/cureus.92519

**Published:** 2025-09-17

**Authors:** Yizhu Yin, Zara Riaz, Rafael Amoro Sanchez, Ahmed Mustafa, Ashkan Eighaei Sedeh

**Affiliations:** 1 College of Medicine, Catholic University of Sacred Heart Rome, Rome, ITA; 2 Internal Medicine, University Hospital Birmingham, Birmingham, GBR; 3 Internal Medicine, Capital Health, Trenton, USA; 4 Internal Medicine, University of Massachusetts, Amherst, USA

**Keywords:** artificial intelligence, chatgpt 4-o, copd (chronic obstructive pulmonary disease), patient-education tool, pulmonary disease

## Abstract

Aim: Chronic obstructive pulmonary disease (COPD) is a leading cause of mortality worldwide. As patient education remains a critical tool in battling chronic conditions, including COPD, a potential role for artificial intelligence (AI) chatbots has taken center stage due to their evolving nature and free availability to the public. However, questions remain on the accuracy and reliability of the information offered by these tools. This study aims to evaluate the accuracy and reproducibility of the responses provided by Chat Generative Pre-trained Transformer (ChatGPT), version 4o (OpenAI, San Francisco, CA), a frequently utilized AI chatbot, to common questions asked by COPD patients.

Materials and methods: A set of 44 patient-centered questions regarding COPD was selected and reviewed by physicians experienced in managing pulmonary disorders to ensure quality and relevance. Each question was submitted to ChatGPT (version 4o) three times by study staff, using separate accounts. A majority response, repeated two times or more, was identified per question and was assessed for accuracy and reproducibility by two physicians experienced in COPD management, using a structured rubric. Responses were classed as accurate (complete responses inline with practice guidelines), partially accurate (omitted some information but relayed key points and did not share false information), or inaccurate (false or misleading information). In case of a disagreement in scoring, a consensus review was performed by a third physician.

Results: The mean accuracy score was moderate (0.61, SD ± 0.14), with 20.5% (9/44) fully accurate, 79.5% (35/44) partially accurate, and no inaccurate responses, which significantly diverges from the hypothetical perfect score (t=-19.14, p<0.001). However, a high reproducibility score was achieved as responses showed consistency across three iterations, for 93.2% (41/44; 95% CI: 81.4-98.3%) of the questions.

Conclusion: While ChatGPT shows consistency in responding to COPD-related queries, it cannot yet serve as a standalone patient-education tool. Even though none of the responses were inaccurate, suggesting a potentially safe resource, oversimplification and omitting key information are major limiting factors leading to partial and incomplete responses, raising concerns for potential misinformation. ChatGPT may serve as a supplementary tool in COPD-related disease management, but its utilization must be accompanied by validation from healthcare professionals.

## Introduction

Chronic obstructive pulmonary disease (COPD) is the third leading cause of morbidity and mortality worldwide, accounting for approximately 3.5 million deaths globally in 2021 [1]. In the United States (U.S.), nearly 16 million adults live with COPD [2], while many undiagnosed cases were not even taken into consideration. The disease imposes a huge economic burden exceeding $49 billion annually in the U.S. as of 2010 [3], leading to increased healthcare expenditure directly and indirectly, as well as loss of productivity. COPD unevenly affects adults among different age groups and occupational groups. The age-adjusted death rate among adults above 75 years old is 855.8 per 100,000, while it stands at 102.5 per 100,000 among ever-employed persons [4]. Employees in food preparation, construction, and mining sectors have significantly higher proportionate mortality ratios (PMRs) [4], indicating elevated risks in certain occupations and revealing the potential to prevent and manage the disease through effective education. Global COPD prevalence was projected to approach 600 million cases by 2050, with a growth of 23% in the number of cases compared with 2020 [5], highlighting an emerging need for enhanced diagnostic and intervention strategies. 

Cigarette smoking remains the essential risk factor for COPD, present in 75% of all cases. Occupational exposures, specifically to dust, fumes, chemicals, and toxins, are demonstrated to be another contributing factor [6]. These risk factors are preventable theoretically; however, effective prevention relies on accessible and qualitative education sources, which are unevenly distributed globally. 

The rapid growth of Generative Artificial Intelligence (GeAI) chatbots has shown thrilling, scalable potential to offer a solution. These tools provide freely available, simultaneous conversational responses to user queries, mimicking human dialogues. While these tools are widely available, their impact in the realm of medical education is understudied. With the accessibility of GeAI to patients outside clinical settings, concerns about its popularity and widespread use are emerging: are these platforms trustworthy enough to complement the face-to-face consultations between patients and physicians? The unregulated nature of GeAI content generation raises critical concerns about its credibility and reliability for unprotected populations who may lack the knowledge to distinguish correct information from misinformation. 

This study aims to evaluate the accuracy and reproducibility of Chat Generative Pre-trained Transformer version 4o (ChatGPT-4o) (OpenAI, San Francisco, CA) responses to COPD patient questions. By examining the alignment of the generated content with current, well-established clinical guidelines, we aim to explore its potential role, advantages, and limitations within the scope of digital health education related to COPD.

## Materials and methods

This is a non-human-subject observational study and did not require approval by the institutional review board.

Question development and selection

An initial set of 70 commonly asked COPD-related questions was gathered by study staff. The questions were evaluated from the perspective of relevance, clarity, redundancy, and educational value by two physicians with an average experience of seven years in treating COPD patients. They were filtered based on what was most commonly encountered in the clinical setting. Questions that were considered less relevant based on clinical experience of caring for patients with COPD were excluded. A final set of 44 questions was selected for this study, and the complete set of these questions is provided in Appendix. 

Generative AI (GeAI) platform selection and query protocol 

Considering its popularity and free public availability, Chat Generative Pre-trained Transformer (ChatGPT, version 4o, OpenAI, San Francisco, CA) was selected as the GeAI platform of choice for this study. Default settings in ChatGPT-4o were used, and no browsing or custom instructions were enabled. To minimize algorithmic bias, three distinct, independently operated ChatGPT accounts were accessed using incognito browsers to avoid AI memory formation. All 44 questions were submitted to each of the three ChatGPT accounts, resulting in 132 responses. To facilitate concise outputs, each query included a prompt not to exceed 100 words. Each generated response was copied onto a data sheet without modification to protect the integrity of the content for the subsequent evaluation and statistical analysis.

Reproducibility analysis 

Three responses generated by GeAI across the three iterations for each question were compared. A response was considered reproducible if the key information was consistent in all three responses. 

Accuracy scoring and evaluation framework

Two physicians based in the U.S. with an average of seven years of experience in diagnosing and managing COPD, and who were blinded to the source of responses, independently reviewed the majority of responses to each question in order to assess response accuracy. A majority response was defined as the response that repeated in terms of conceptual consistency at least two of the three times that each question was put through GeAI.

Data analyses

Data analyses, including descriptive statistics, inter-rater reliability, one-sample t-test, etc., were conducted using Stata Statistical Software: Release 19 (StataCorp LLC, College Station, TX). Statistical significance was defined as a two-sided p-value of less than 0·05. Responses were evaluated using the three-tiered accuracy rubric shown in Table 1.

**Table 1:** Accuracy scoring rubric.

Score	Definition
1	Response is clinically accurate, complete and guideline-concordant.
0.5	Response is generally correct but omits key information or lacks clinical nuance.
0	Response contains factual errors or misleading content.

Inter-rater variability and consensus review

**Figure 1:**
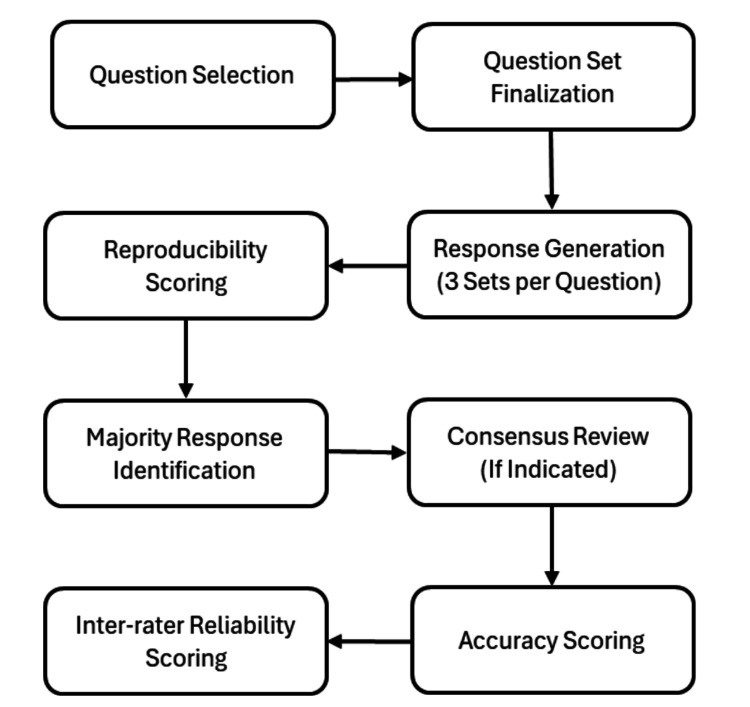
Study workflow.

**Figure 2:**
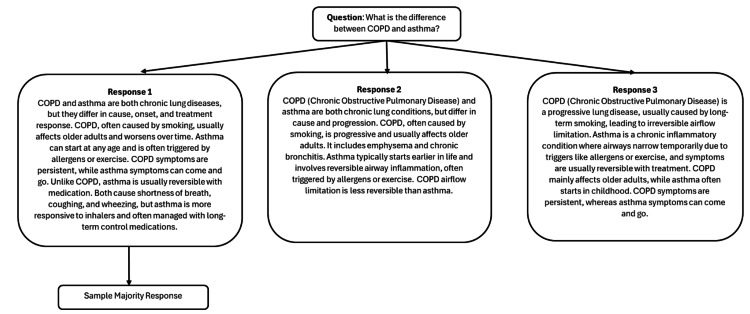
Majority response selection algorithm.

In case of a disagreement between the scores given by each of the two reviewers, a third reviewer with seven years of experience performed a consensus review of the said responses. The study workflow is illustrated in Figure 1. A copy of a sample question, three generated responses, and the selected majority response are presented in Figure 2.

## Results

Reproducibility of generative AI (GeAI) responses 

Of the 44 generated responses, 93.2% (41/44, 95% CI: 81.4%-98.3%) were consistent across all three iterations, demonstrating the steady performance of GeAI in regenerating similar responses to the same query and a high reproducibility score. The remaining 6.8% (3/44) omitted key clinical information or varied in their focus on different aspects.

Inter-rater reliability and consensus 

A disagreement was present between the two independent reviewers in scoring 27.3% (12/44) of the responses. These questions and answers underwent a blinded consensus review by a third physician. In all cases, the scores by the third physician matched either one of the primary reviewers and were selected as the final score. After resolution, the final classification consisted of nine partially accurate (score=0.5) and three fully accurate (score=1) responses, and none of the responses were rated as inaccurate (score=0).

A moderate agreement between the two primary reviewers was identified, with a Cohen's Kappa score of 0.62 (95% CI: 0.42-0.82). The major disagreements occurred in differentiating partially and fully accurate responses, particularly when the generated content included correct terminology without elaboration or sufficient explanation. 

Accuracy of majority responses 

**Figure 3:**
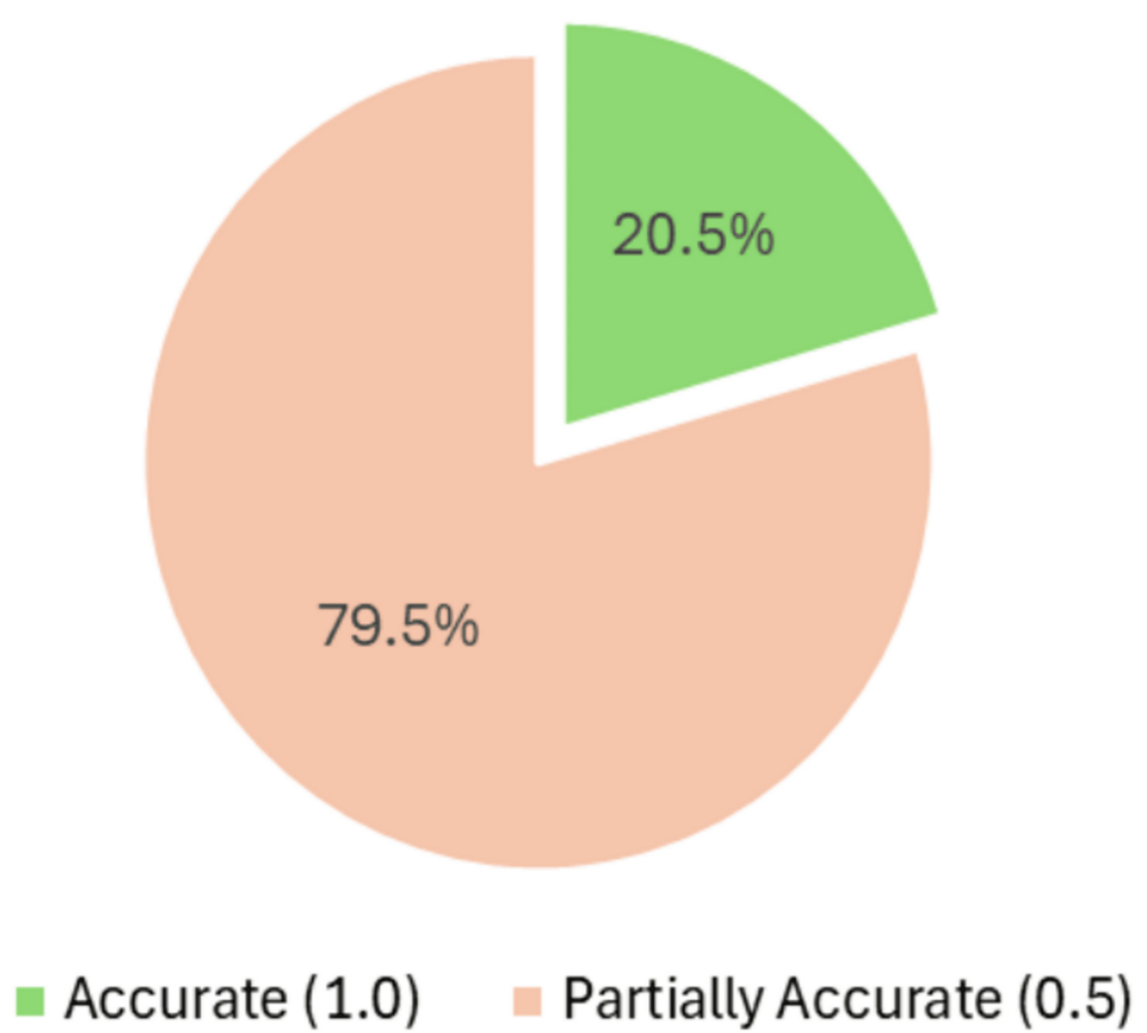
Accuracy distribution of ChatGPT responses to COPD questions. ChatGPT: Chat generative Pre-trained Transformer, COPD: chronic obstructive pulmonary disease.

As presented in Figure 3, across the majority of responses, 20.5% (9/44) were scored as accurate (score=1), while a notably greater number of responses, 79.5% (35/44), were found to be partially accurate (score=0.5). No responses were rated as inaccurate, suggesting the absence of misleading or potentially harmful information in COPD-related patient educational content generated by GeAI.

The mean accuracy score across all items was 0.61 ± 0.14. A one-sample t-test was performed to compare the observed mean score to the hypothetical ideal score of 1.0 (perfect accuracy). The result proved to be highly statistically significant (t=-19.14, df=43, p<0.001), confirming a meaningful difference between GeAI responses and the gold-standard clinical guidelines.

As demonstrated in Table 2, response accuracy was further analyzed across five thematic categories of COPD-related questions: (1) disease overview and epidemiology, (2) diagnosis and disease course, (3) treatment, (4) prevention, and (5) monitoring and rehabilitation. Mean accuracy scores varied modestly by category, with the lowest average score for treatment-related questions.

**Table 2:** Category-wise response accuracy.

Category	Total questions	Accurate (1.0)	Partially accurate (0.5)	Inaccurate (0)	Mean score
Disease overview and epidemiology	9	3	6	0	0.61
Diagnosis and disease course	9	2	7	0	0.56
Treatment	11	1	10	0	0.55
Prevention	11	2	8	0	0.60
Monitoring and rehabilitation	5	1	4	0	0.60
Total/overall	44	9	35	0	0.61

## Discussion

This study assessed the reproducibility and clinical accuracy of responses generated by GeAI to frequently asked questions (FAQs) related to COPD. The majority of responses were reproducible, but only partially accurate, posing a risk related to the incomplete and oversimplified clinical content. 

The findings of this study are aligned with other literature evaluating the reliability of large language models (LLMs) in clinical practice and patient education. In a study conducted by Odabashian et al., ChatGPT-3.5 was evaluated on commonly asked oncology-related questions, with only 56% accurate responses; the majority were categorized as “incomplete but directionally correct” [7]. Similarly, Gilson et al. found that while ChatGPT demonstrated strong performance on answering standardized medical licensing examination questions, its accuracy markedly reduced when applied to open-ended clinical cases [8]. In another study on inflammatory bowel diseases, Gravina et al. failed to differentiate between Crohn's disease and ulcerative colitis [9]. In another study, in the context of pediatric familial Mediterranean fever, La Bella et al. [10] concluded that ChatGPT-4o is relatively accurate in responding to queries regarding treatment, whilst misinterpreting diagnostic criteria, for example, the absence of genetic factors (e.g., Mediterranean fever (MEFV) gene mutations). Similar omissions in diagnosis were presented in our research, such as omitting spirometry in the early diagnostic explanation. Additionally, when studying responses generated by various LLMs, including ChatGPT, in the context of endometriosis patients' frequently asked questions (FAQs), Cohen et al. found that LLMs are less effective in answering treatment-related questions, while performing well in responding to symptom-related questions [11]. It is important to note that while some studies, including one by Ayers et al. [12] on the role of ChatGPT as an educational tool in the area of preventive health, had a significantly higher accuracy score, the majority of such studies evaluated questions of low complexity and general wellness, rather than specific diseases. 

What distinguishes our study is its focus on chronic disease education from the perspective of patient comprehension, a domain where partial facts may be just as problematic as incorrect information. For example, suppose ChatGPT explains that inhalers are the cornerstone of COPD treatment but omits the importance of proper technique and adherence. In that case, a patient may believe they are fully informed while still using the medication ineffectively, undermining disease control and outcomes. While ChatGPT-4o performed well in avoiding outright misinformation, with no response scoring 0, it frequently failed to meet the clinical detail required for safe autonomous use. These information gaps may propagate subtle misunderstandings that impact patient adherence, self-management, or health-seeking behavior. 

While in-person consultation requires Health Insurance Portability and Accountability Act compliance and is closely monitored by the host institution, the use of GeAI blurs the lines between patients’ information ownership and data accessibility due to the current lack of regulations. This is particularly important, given that healthcare systems are vulnerable to cyberattacks [13]. First, this study solely focused on COPD-related questions, which cannot necessarily be applied across a broad dimension of diseases; results may differ in other disease domains, especially those with less codified guidelines or higher clinical ambiguity. This necessitates further studies to explore the accuracy and reproducibility of responses generated by ChatGPT. Second, the responses were evaluated in English only and in the context of the U.S. healthcare guidelines. This limits the assessment of compliance with clinical guidelines in other countries and cross-cultural generalizability. Third, while our accuracy rubric was structured and inter-rater validated, the scoring process remains inherently subjective, and different reviewer backgrounds may yield different interpretations. Importantly, the need for consensus scoring in 27.3% of questions illustrates the blurred line between “correct enough” and “clinically sufficient.” Limiting the responses generated to 100 words possibly played a role in resulting in incomplete and partially accurate responses; however, this constraint was intentionally applied to prevent ChatGPT-4o from producing excessively long answers and to evaluate its ability to generate concise, practical responses suitable for real-world patient education. Furthermore, it is important to note that having a high reproducibility of partially accurate responses could inadvertently reinforce incomplete information if widely deployed without oversight, underscoring the need for clinician-guided use.

Currently, there is no widely adopted guideline in the implementation of GeAI in clinical practice [14]; further research on comparing GeAI with established clinical guidelines across diverse diseases is required. As GeAI continues to evolve, its integration into patient education must be guided by in-depth evaluation and ethical foresight. Future studies should examine performance across multiple languages, literacy levels, and disease types, and should assess real-world impacts on patient understanding, behavior, and clinical outcomes. 

## Conclusions

While ChatGPT responses were highly reproducible but often incomplete, none contained false information, which is a clinically reassuring finding. This study recommends cautious application of GeAI in the healthcare setting, in conjunction with human supervision and not as a standalone tool. Our findings suggest that ChatGPT-4o cannot yet be considered a reliable standalone tool for unsupervised patient education in chronic diseases, using COPD as an example. While it may serve as an adjunct within physician-guided interactions or curated platforms, its use should be coupled with real-time oversight. The optimal role of GeAI may be as a supportive adjunct within structured, clinician-guided environments or curated digital platforms where content can be reviewed and corrected in real time. Future improvement of GeAI tools, with expanded contextual reasoning and more transparent accountability mechanisms, may hold greater promise. Until then, the deployment of such models in patient-facing applications should prioritize safety and clarity, along with compatibility with evidence-based guidelines. Further research across a wider range of diseases, languages, and healthcare settings is essential to better evaluate the utility, risks, and ethical implications of GeAI in medicine.

## Appendices

Appendix: The final question list

1.  What is chronic obstructive pulmonary disease (COPD)?

2.  How is a diagnosis of COPD established?

3.  What are the primary risk factors associated with COPD?

4.  Is COPD equivalent to emphysema?

5.  What is the difference between COPD and asthma?

6.  How can I be diagnosed with COPD if I have never smoked?

7.  What are the clinical signs and symptoms of COPD?

8.  What pathological changes occur in the lungs of a person with COPD?

9.  How does alpha-1 antitrypsin deficiency-related COPD differ from typical COPD?

10.  Is COPD a reversible condition?

11.  Is COPD considered a terminal illness?

12.  Is there a genetic component to COPD?

13.  What is the relationship between smoking and the development of COPD?

14.  What strategies can help prevent the onset of COPD?

15.  How can I minimize my risk of lung infections if I have COPD?

16.  Which environmental factors can trigger COPD exacerbations?

17.  What are the available treatment options for managing COPD?

18.  What are the best practices for managing COPD at home?

19.  What modifications should I make in my home environment to support COPD management?

20.  What workplace accommodations can help improve my condition with COPD?

21.  What should I prepare for when planning air travel with COPD?

22.  Does having COPD increase the risk of developing other medical conditions?

23.  What daily medications are recommended for someone with COPD?

24.  Which quick-relief or rescue medications should I use when experiencing shortness of breath with COPD?

25.  What treatment-related side effects should I monitor as a patient with COPD?

26.  How can I determine when my inhalers are running low if I have COPD?

27.  When should I use a nebulizer instead of an inhaler in the management of COPD?

28.  What is lung volume reduction surgery, and how is it used in COPD treatment?

29.  What are the potential side effects of oxygen therapy in patients with COPD?

30.  When is oxygen therapy indicated for patients with COPD?

31.  Under what circumstances is lung volume reduction surgery appropriate in COPD management?

32.  Should oxygen therapy be administered continuously in patients with COPD?

33.  What are the indicators of disease progression in COPD?

34.  What should I do if I feel I am not breathing adequately due to COPD?

35.  Under what circumstances should I contact my healthcare provider if I have COPD?

36.  When should I seek emergency medical care for COPD symptoms?

37.  What factors are most likely contributing to my COPD symptoms?

38.  What typically causes episodic flare-ups in COPD symptoms?

39.  Why does COPD progress over time even with proper management?

40.  How should I manage panic attacks that occur in the context of COPD?

41.  When can pulmonary rehabilitation be beneficial for managing COPD?

42.  Are there any activity restrictions or precautions I should follow as a person with COPD?

43.  Why is proper nutrition important when living with COPD?

44.  Are there specific dietary guidelines that can help control COPD symptoms?
